# Downbeat nystagmus and progressive ataxia in adults: consider Chiari malformation type 1

**DOI:** 10.1055/s-0043-1771171

**Published:** 2023-09-29

**Authors:** Breno Kazuo Massuyama, Thiago Cardoso Vale, Flávio Moura Rezende Filho, Orlando Graziani Povoas Barsottini, José Luiz Pedroso

**Affiliations:** 1 Universidade Federal de São Paulo, Departamento de Neurologia, Unidade de Ataxia, São Paulo SP, Brazil.; 2 Universidade Federal de Juiz de Fora, Departamento de Medicina Interna, Juiz de Fora MG, Brazil.

Downbeat nystagmus (DBN) is present in between 4 and 6% of patients with Chiari malformation type 1 (CM1). It is present in primary gaze and is characterized by a pathological phase which drifts the eyes in the upward direction followed by a downward quick-phase. 1 2 3 Chiari malformation type 1 is one of the most prevalent craniocervical junction abnormalities 4 and is more frequent in Northeastern Brazilians, due to pre-historic ancestors (previously thought to be caused by the Dutch colonization). 5 The herniation of cerebellar tonsils through the foramen magnum is radiologically characterized by the distance between the apex of the odontoid and Chamberlain line of at least 5 mm. 6

**Figure 1:**
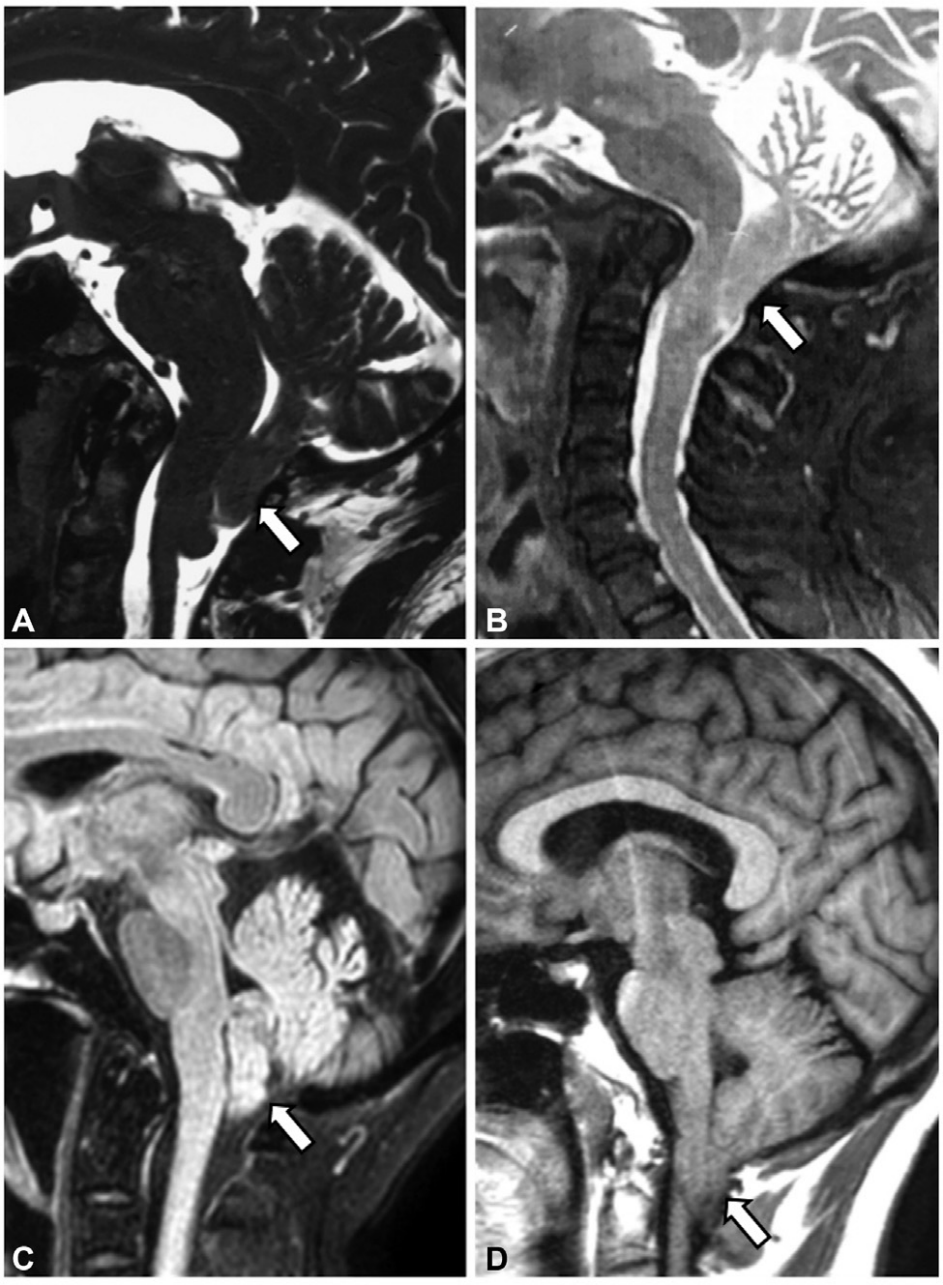
(Case A-D) Cranio-cervical MRI showing Chiari malformation type 1 in the four patients with progressive ataxia and downbeat nystagmus, which is defined by herniation of cerebellar tonsils through the foramen magnum (located at least 5 mm below this structure) (arrows).

We present a four-case series of CM1 patients who developed progressive cerebellar ataxia and DBN. Brain magnetic resonance imaging (MRI) revealed the abnormal projected cerebellar tonsils. Evaluation of the posterior fossa through brain imaging looking for structural abnormalities such as CM1 is mandatory in cases of progressive ataxia combined with DBN, headache, and pyramidal signs ( Video 1 and Fig. 1 ).

**Video 1** Video of the patients with progressive ataxia and downbeat nystagmus related to Chiari malformation type 1.

